# Price and Healthfulness of Snacks in 32 YMCA After-School Programs in 4 US Metropolitan Areas, 2006-2008

**DOI:** 10.5888/pcd9.110097

**Published:** 2012-01-12

**Authors:** Rebecca S. Mozaffarian, Analisa Andry, Rebekka M. Lee, Steven L. Gortmaker, Jean L. Wiecha

**Affiliations:** Harvard Prevention Research Center, Department of Society, Human Development and Health, Harvard School of Public Health; Brigham and Women’s Hospital, Division of General Internal Medicine and Primary Care, Boston Massachusetts; Harvard School of Public Health, Boston, Massachusetts; Harvard School of Public Health, Boston, Massachusetts; University of Massachusetts Boston, Boston, Massachusetts

## Abstract

**Introduction:**

A common perception is that healthful foods are more expensive than less healthful foods. We assessed the cost of beverages and foods served at YMCA after-school programs, determined whether healthful snacks were more expensive, and identified inexpensive, healthful options.

**Methods:**

We collected daily snack menus from 32 YMCAs nationwide from 2006 to 2008 and derived prices of beverages and foods from the US Department of Agriculture price database. Multiple linear regression was used to assess associations of healthful snacks and of beverage and food groups with price (n = 1,294 snack-days). We identified repeatedly served healthful snacks consistent with Child and Adult Care Food Program guidelines and reimbursement rate ($0.74/snack).

**Results:**

On average, healthful snacks were approximately 50% more expensive than less healthful snacks ($0.26/snack; SE, 0.08; *P* = .003). Compared to water, 100% juice significantly increased average snack price, after controlling for other variables in the model. Similarly, compared to refined grains with trans fats, refined grains without trans fat significantly increased snack price, as did fruit and canned or frozen vegetables. Fresh vegetables (mostly carrots or celery) or whole grains did not alter price. Twenty-two repeatedly served snacks met nutrition guidelines and the reimbursement rate.

**Conclusion:**

In this sample of after-school programs, healthful snacks were typically more expensive than less healthful options; however, we identified many healthful snacks served at or below the price of less healthful options. Substituting tap water for 100% juice yielded price savings that could be used toward purchasing more healthful foods (eg, an apple). Our findings have practical implications for selecting snacks that meet health and reimbursement guidelines.

## Introduction

Given the dual challenges of the obesity epidemic and the current economic recession, identifying healthful beverages and foods that are reasonably priced is more important than ever. US after-school programs serve 8.4 million children annually; thus, they can substantially affect the nutritional value of snacks offered to children (1). Overweight and obesity prevalence is high among US children, particularly lower-income and racial/ethnic minority populations (2), who are also more likely to attend after-school programs (3). Populations living in low-income and minority areas are less likely to have access to healthful, affordable food (4), further emphasizing the importance of serving healthful snacks in the after-school setting. Snacking frequency among children has increased (5); thus, each snacking occasion is an opportunity to influence health.

Healthful foods are often perceived to be more expensive than less healthful foods (6). Because after-school programs typically operate on a limited budget, healthful, affordable snacks should be identified. From 2009 to 2010, the US Department of Agriculture (USDA) Child and Adult Care Food Program (CACFP) reimbursed participating after-school programs $0.74 per snack, including food and beverage (7). However, the contributions of different beverages and foods to total daily snack price in after-school programs, and relationships between price and healthful characteristics, have not been evaluated. These issues are especially relevant for after-school programs that wish to serve healthful snacks while meeting CACFP nutrition guidelines and reimbursement rates.

The objective of this study was to compare the prices of healthful and less healthful snacks and to analyze the independent contributions of individual beverages and foods to total daily snack price during 9 consecutive weeks at 32 YMCA after-school programs in 4 US metropolitan areas. We hypothesized that, on average, snacks that met healthful eating standards would cost more. As a secondary objective, we investigated whether any repeatedly served (≥5 times) after-school snacks met healthful eating standards, CACFP nutrition guidelines, and reimbursement rates. We hypothesized that some snack combinations would meet these criteria.

## Methods

### Setting and design

We prospectively collected daily snack menu data from 32 YMCA after-school sites in 4 metropolitan areas nationwide (in the Pacific Northwest, Midwest, South, and East) during 2 years (fall of 2006 through spring of 2008). YMCA sites were participating in the YMCA/Harvard Afterschool Food and Fitness Project, a quasi-experimental intervention to promote physical activity and healthful eating among children aged 5 to 12 years attending after-school programs. Because the intervention was implemented at 16 sites in October 2006, we used baseline data (9 consecutive weeks; 1,294 snack-days) to investigate the costs of beverages and foods served. We used all of the data we collected over the 2-year period (9,787 snack-days) to assess repeatedly served snacks that met healthful eating standards, CACFP nutrition guidelines, and the CACFP reimbursement rate to best estimate the number and variety of snack combinations. The average percentage of white children and median family incomes were obtained by using program site zip codes. The Committee on Human Subjects at the Harvard School of Public Health approved the study.

### Snack data collection

For each snack day throughout the 2-year period, YMCA staff recorded all beverages and foods served. Staff completed electronic or paper reports and submitted them monthly to the Harvard School of Public Health Prevention Research Center. Menus provided details on specific types and brands of beverages and foods served, including types of milk, juice, and water, and whether fruits and vegetables were fresh, canned, dried, or frozen. For the primary objective, data were available from 98.2% of possible snack-days during the 9-week baseline period.

### Price determination

To determine nationally representative prices of each beverage and food served, we used the USDA Center for Nutrition Policy and Promotion (CNPP) price database (8). The database contains data on 4,634 individual beverages and foods identified from the National Health and Nutrition Examination Survey; methods for price determination are described elsewhere (9).

We matched each beverage and food item reported by programs to the most similar item in the CNPP database. Brand-specific items are typically not included in this database; we matched these items to the most similar generic item (eg, "Cheez-it crackers" matched to "cheese crackers"). Of the 54 brand-specific items reported by staff, 47 were not in the database.

We used the 2003-2004 national price average per 100 g for each beverage and food. We calculated per-serving prices on the basis of standard serving sizes as specified on product or grocery store websites, labels, or the USDA's Food Guide Pyramid (10). To enable comparison to the 2009-2010 CACFP reimbursement rates, we adjusted prices for inflation using the Bureau of Labor Statistics Food and Beverage Consumer Price Indices for All Urban Consumers (11,12) to correspond to the mid-fiscal year (December 2009). Factoring in bulk price of disposable cups (www.costco.com), we assigned tap water a price of $0.03 per serving. Using a nationally representative price database and adjusting for inflation enabled us to investigate average prices independent of regional differences and to compare prices with current reimbursement rates.

### Nutrient data and classification of foods and beverages

As described in detail elsewhere (13), we collected calorie, fiber, and ingredient information for each beverage and food from product and grocery store websites (www.peapod.com), package labels, and for generic items/unreported brands, from the USDA Nutrient Database (14).

We classified beverages into 5 categories: water, 100% juice, 1% or 2% unflavored milk, 1% or 2% flavored milk, or sugar-sweetened beverages. Neither skim nor whole milk was served. YMCA staff were instructed to report serving water only if they served it at the snack table (ie, not if available from a fountain or cooler only).

We classified foods into 4 major categories: grains (eg, breads, crackers, cereals, tortillas), vegetables, fruits, and protein foods (eg, cheese, yogurt, peanut butter, beans, nuts, meat, tuna). We classified grains and protein foods according to CACFP's creditable food guidelines (15). We further subcategorized grains into refined grains with and without trans fat and whole grains with and without trans fat. We classified grains with a carbohydrate-to-fiber ratio of ≤10:1 as whole grains (16) and identified trans fat by the presence of "partially hydrogenated oil" in the ingredient list. We subcategorized vegetables into canned or frozen and fresh, and fruits into canned, dried, or frozen and fresh. We classified mixed dishes and combination foods into more than 1 food group (eg, vegetable noodle soup as vegetable and grain).

### Healthful snack guidelines

To evaluate healthful snacks, we used Environmental Standards for Healthy Eating (ESHE), guidelines adapted from a YMCA/Harvard collaboration to help staff for out-of-school programs select and serve healthful snacks (17). The ESHE are guidelines for improving after-school snack quality by meeting 5 simple standards: do not serve sugar-sweetened beverages, serve water every day, serve a fruit and/or vegetable every day, do not serve foods with trans fat, and when serving grains (such as bread, crackers, and cereals), serve whole grains (17).

CACFP guidelines provide reimbursement for snacks served at after-school programs in low-income areas. For participating after-school programs to qualify for reimbursement, snacks must contain at least 2 of 4 components: fluid milk; meat or meat alternate (eg, beans, cheese); vegetable, fruit, or full-strength fruit juice; or whole grain or enriched bread or cereal (18). The 2009-2010 CACFP reimbursement rate was $0.74 per snack. Only 2 programs in this analysis participated in CACFP; we use the reimbursement rate as a price guideline so that our findings may be applied to participating programs.

### Statistical analysis

To determine price differences between healthful and less healthful snacks, we included an indicator variable for ESHE achievement in the regression ("yes" if all 5 standards met; otherwise "no"). We performed multiple linear regression to assess associations between total snack price as the dependent variable and different beverages and foods as independent variables. We used a Q-Q plot to assess normality of snack prices. Beverages were considered mutually exclusive indicator variables as classified above; water was the reference. Foods were considered indicator variables as classified above; refined grains containing trans fat were the reference. These references were chosen because they were the most inexpensive and frequently served in the beverage and food categories. No sites served sugar-sweetened beverages or whole grains with trans fats; thus, we could not evaluate these variables. We evaluated beverages and foods simultaneously in the model and further adjusted for each YMCA association by metropolitan area (4 categories) and the total number of food items in the snack (range, 1-5).

To assess which repeatedly served snacks met ESHE and CACFP guidelines and cost less than $0.74, we summed the number of times snack combinations meeting these criteria were served during the 2-year period. To determine frequency, we combined average values of similar beverage and food items (eg, 1% with 2% unflavored milk). Assuming tap water is safe, it can be served at a low price; we added the price of water to each snack at $0.03 per serving to account for disposable cups. To maximize healthfulness, we included unflavored milk only. Total snack price was calculated by summing adjusted prices of beverages and foods that made up each snack.

We used SAS 9.2 (SAS Institute, Inc, Cary, North Carolina) to account for clustering of snacks at the YMCA site level. Significance was set at *P* < .05.

## Results

The mean price of each daily snack served ranged from $0.47 in the Midwest and Northeast metropolitan areas to $0.78 in the Pacific Northwest; the overall average was $0.57 per snack (Table 1). Daily snack prices ranged from $0.04 (oatmeal cookie only) to $2.47 (apple slices, granola, yogurt, orange, water). Patterns of snack offerings varied among sites; some served snacks that varied weekly; others served weekly cycles of the same menu. The average percentage of white children and median annual incomes in the areas where these sites were located were higher than the US average.

Only 17% of snacks served met all ESHE guidelines (Table 2). Snacks that met ESHE were approximately 50% more expensive than snacks that did not ($0.26/snack; SE, 0.08; *P* = .003). Approximately half of all snacks contained 1 food item; snack price increased with the number of foods served. Tap water was served on nearly half of snack-days.

A grain was served on 75% of snack-days; whole grains were served on only 5%. Cheerios ($0.23/serving) and Triscuit crackers ($0.11/serving) were the most frequently served whole grains. Refined grains containing trans fat were served on 42% of snack-days; refined grains without trans fat, on 29%. The most frequently served refined grains with trans fat were saltine-type crackers ($0.07/serving) and graham crackers ($0.26/serving). The most frequently served refined grains without trans fat were pretzels and tortilla chips ($0.20/serving each). Vegetables were served on only 18% of snack-days and fruits on 27%. The most frequently served vegetables were carrots ($0.18/serving) and celery ($0.12/serving); the most frequently served fruits were applesauce ($0.23/serving), apples ($0.36/serving), and bananas ($0.22/serving). On average, the least expensive food categories were refined grains with trans fat ($0.21/serving; 40 foods) and fresh vegetables ($0.22/serving; 14 foods). The most expensive food categories were canned or frozen vegetables ($0.46/serving; 16 foods) and protein foods ($0.40/serving; 51 foods).

Snacks including beverages other than water were more expensive, ranging from $0.10/snack for 1% or 2% unflavored milk to $0.21/snack for 100% juice (Table 3). Snacks including refined grains without trans fat were more expensive than those with trans fat, yet snacks containing whole grains without trans fat were not different in price. Compared to snacks containing refined grains with trans fat, snacks including canned or frozen vegetables were more expensive; in contrast, snacks including fresh vegetables (mostly carrots and celery) were not. Snacks including canned, dried, or frozen fruit, fresh fruit, or protein foods were more expensive than snacks containing refined grains with trans fat.

When the ESHE achievement variable was included in the regression, healthful snacks that met the guidelines were significantly more expensive than snacks that did not ($0.22/snack; SE, 0.07; *P* = .003) (data not shown).

Twenty-two after-school snacks were repeatedly served, met ESHE and CACFP guidelines, and cost less than $0.74 per snack (Table 4). The most common snack was fresh fruit with 1% or 2% unflavored milk and water. Other low-priced healthful snacks were canned pineapple in light syrup, 1% milk, and water, and carrots, hummus, and water. Total calories ranged from 71 to 370 kcal per snack, and dietary fiber from 1.0 to 4.4 g per snack.

## Discussion

Our data demonstrate that healthful snacks that meet the ESHE guidelines are on average more expensive than less healthful snacks. This difference was largely driven by fruits and canned or frozen vegetables, which are higher-priced foods consistent with ESHE standards. Our findings suggest useful strategies to increase the healthfulness of snacks without large price increases. These strategies include serving snacks with low-priced fresh vegetables, replacing refined grains with whole grains, and replacing 100% juice with tap water.

Our analysis identified repeatedly served snacks that met guidelines for healthful eating and CACFP nutrition guidelines and reimbursement rates. Many of these snacks contained lower-priced fruits and protein foods, indicating that programs could budget to occasionally include more expensive healthful foods rather than excluding them entirely. Although many potential snack combinations could be theoretically constructed on the basis of price and nutrition data alone, our analyses were based on snacks actually served, inherently incorporating issues of availability, feasibility, and acceptance in real-world situations.

To our knowledge, no prior investigation has prospectively evaluated the determinants of after-school snack prices. Tools for pricing and planning healthful snacks have been developed for after-school staff (19,20), indicating interest in guidance on this area.

Evidence from economic analyses is consistent with our findings that less healthful foods are generally less expensive (21-23). Our findings for fruits and canned or frozen vegetables are consistent with these analyses; however, they also indicate that the perception that more healthful foods are always more expensive is oversimplified. Studies often evaluate food prices per calorie rather than per serving, biasing results toward finding lower prices for high-calorie, energy-dense foods that would also generally be less healthy (21-23). Such calorie-based metrics can create a circular argument, implicitly handicapping lower-calorie foods such as fruits and vegetables. Shifting from valuing foods strictly on a per-calorie basis toward foods that are satiating and nutritious would provide a more comprehensive view of the healthfulness of foods on which to evaluate price.

After-school programs would benefit from allocating money spent on less healthful beverages and foods toward more healthful options. Assuming tap water is safe to drink at after-school programs, serving water would save money that could be spent on even more healthful food options for snacks such as whole fruits, while still meeting price targets. Whole fruit is preferable to 100% juice because of its higher fiber content and effects on satiety and also because it requires a longer time to consume the same number of calories (24). On the basis of our data, programs could add a banana and tap water to the snack for the same price as 100% apple juice ($0.24). Similarly, tap water and cheese slices instead of 1% chocolate milk would save $0.03 per serving and reduce sugar, while providing other nutrients. Within food categories, Cheerios and Triscuits were examples of whole grains that were less expensive than some refined grains with trans fat (eg, graham crackers). A recent study found that healthful foods that did not increase price were available within food categories (25), further confirming the feasibility of this strategy. Moreover, similar to our definition of healthful foods, this study used metrics other than energy density alone to define healthful options (eg, no trans fat or white flour).

Little standardized guidance is offered for snacks in after-school programs, nor for upper or lower limits for total snack calories (26). Programs generally must adhere to varying standards based on their specific local program, organization, or state agency. CACFP guidelines provide no direction beyond requirements to serve at least 2 of 4 components; by these standards, a reimbursable after-school snack could be Pop-Tarts (a grain) and milkshakes (a milk product). Development of standardized guidelines merits consideration. The National Afterschool Association adopted voluntary quality standards in 2011, a notable shift toward addressing this need (26). Reimbursement guidelines can also complicate snack choices. For example, CACFP specifies that fruits and vegetables are reimbursable only in serving sizes of at least three-fourths of a cup (10). Because some whole fruits count as less than a three-fourths cup serving (eg, a small apple or raisins), these reimbursement guidelines lead programs to also serve 100% juice to meet the guideline. After-school programs would have greater flexibility, nutritionally and financially, if they received reimbursement for standard age-appropriate serving sizes of fruits and vegetables.

This study has several limitations. Price analyses did not account for labor, preparation, or overhead costs (except price of water cups). We expect that such price differences would be small for the types of snacks served at these programs; for instance, repeatedly served snacks often included 1 food item requiring minimal or no preparation (eg applesauce, crackers, or banana). Food prices and availability vary by metropolitan area (27), and some fruits and vegetables are less expensive in season than out-of-season. However, many frequently served fruits (eg, apples, bananas, applesauce) and vegetables (eg, carrots, celery) are available year-round with little seasonal price fluctuation (28), making these good choices for after-school programs.

YMCAs in the Pacific Northwest metropolitan area served the widest variety of fresh fruits and vegetables; it is unclear if this was because of availability or because they served more expensive snacks. Additionally, some programs have vendor contracts, limiting control over foods served.

Menus were not independently validated. We collected data on beverages and foods served, not what was actually consumed; more research is needed to determine what kinds of healthful, inexpensive snack combinations children enjoy. Trans fat and nutrient information were obtained from 2006 to 2007; product formulations may have changed. These data do not represent snacks or prices of all after-school programs. Programs were located in areas with higher median income compared to the national median and lower percentages of racial/ethnic minority children than typical nationally, suggesting that this sample of YMCA after-school programs could have served more healthful and expensive snacks than programs in less affluent areas. Furthermore, these programs were situated within associations that had been part of a larger wellness initiative for several years. Nevertheless, our findings represent 32 sites in 4 US metropolitan areas in the largest private, nonprofit after-school provider nationwide and may be considered a reasonable estimate of average snack quality and prices in after-school programs.

Although healthful snacks are more expensive overall than less healthful snacks, our findings demonstrate that a range of healthful foods and snack combinations can be served at a similar or lower price than less healthful options. In light of the obesity epidemic and the increasing number of children attending after-school programs (1), these findings may help programs to purchase and offer more healthful, affordable snacks.

## Figures and Tables

**Table 1:** Characteristics of YMCA After-School Associations Participating in the YMCA/Harvard Afterschool Food and Fitness Project, United States, 2006

**Metropolitan YMCA Association Area**	No. of After-School Sites	White Children Aged 5-14 y in Zip Code of Site, %^a^	Annual Household Income in Zip Code of Site, Median, $^b^	No. of Snack-Days Analyzed	Daily Price of Food and Beverage Snacks, Mean (SD), $^c^	Price Range of Food and Beverage Snack, $^c^
Northeast	8	72.5	47,375	345	0.47 (0.19)	0.04-1.36
Pacific Northwest	10	66.7	63,985	389	0.78 (0.39)	0.15-2.47
South	8	76.8	45,576	328	0.51 (0.13)	0.25-0.64
Midwest	6	91.4	44,012	232	0.47 (0.09)	0.23-0.76
Total	32	76.8	50,237	1,294	0.57 (0.28)	0.04-2.47

Abbreviation: SD, standard deviation.

a Zip code data derived from 2000 census; 61.3% of US children aged 5-14 are white.

b Zip code data derived from 2000 census; the 2000 annual median household income in the United States was $41,994.

c Inflation-adjusted to December 2009 prices.

**Table 2:** Characteristics of Snacks Served at 32 YMCA After-School Programs During 9 Consecutive Weeks (n = 1,294 Snack-Days), United States, 2006

**Characteristic**	No. of Days Served (%)	No. of Unique Beverage or Food Items Served	Price, Mean (SD), $^a^
**Environmental Standards for Healthy Eating^b^**			
Did not meet all standards	1,074 (83.0)	NA	0.53 (0.21)
Met all standards	220 (17.0)	NA	0.79 (0.44)^c^
**No. of food items served/snack**			
1	679 (52.5)	NA	0.49 (0.14)
2	438 (33.8)	NA	0.65 (0.33)
3	153 (11.8)	NA	0.66 (0.37)
4	18 (1.4)	NA	0.99 (0.43)
5	6 (0.5)	NA	1.36 (0.08)
**Beverages served^d^**			
Water	599 (46.3)	1	0.03 (0)
100% juice	415 (32.1)	6	0.26 (0.08)
1% or 2% unflavored milk	355 (27.4)	2	0.19 (0.003)
1% or 2% flavored milk	87 (6.7)	2	0.34 (0.12)
Sugar-sweetened beverages	0	0	NA
**Foods served^d^**			
All grains	975 (75.3)	102	0.25 (0.21)
Refined grains with trans fat	545 (42.1)	40	0.21 (0.19)
Refined grains without trans fat	369 (28.5)	48	0.28 (0.22)
Whole grains with trans fat	0	0	NA
Whole grains without trans fat	61 (4.7)	14	0.27 (0.18)
All vegetables	226 (17.5)	30	0.35 (0.28)
Canned or frozen vegetables	65 (5.0)	16	0.46 (0.34)
Fresh vegetables	161 (12.4)	14	0.22 (0.09)
All fruits	354 (27.4)	32	0.33 (0.17)
Canned, dried, or frozen fruits	145 (11.2)	17	0.33 (0.14)
Fresh fruits	209 (16.2)	15	0.34 (0.21)
Protein foods^e^	286 (22.1)	51	0.40 (0.30)

Abbreviations: SD, standard deviation; NA, not applicable.

a Inflation-adjusted to December 2009 prices.

b The Environmental Standards for Healthy Eating are the following: Do not serve sugar-sweetened beverages; serve water every day; serve a fruit and/or vegetable every day; do not serve foods with trans fat; and when serving grains (such as bread, crackers, and cereals), serve whole grains (19).

c Difference between $0.53 and $0.79 is significant ($0.26/snack; SE, 0.08; *P* = .003).

d Total exceeds 100% because the servings of each item are not mutually exclusive.

e Includes cheese, yogurt, peanut butter, beans, nuts, meat, tuna.

**Table 3:** Price Differences for 1 Complete After-School Snack^a^ at 32 YMCA After-School Sites, by Metropolitan Area and Snack Characteristics, 2006-2008^b^

**Characteristic**	Price Difference, Mean (SE), $^c^	*P* Value^d^
**Metropolitan YMCA Association Area**		
Northeast	1 [Reference]	NA
Pacific Northwest	0.17 (0.03)	<.001
South	0.03 (0.02)	.18
Midwest	−0.004 (0.03)	.89
**No. of food items served/snack**	0.05 (0.02)	.007
**Beverages served**		
Water	1 [Reference]	NA
100% juice	0.21 (0.03)	<.001
1% or 2% unflavored milk	0.10 (0.03)	.002
1% or 2% flavored milk	0.18 (0.02)	<.001
Sugar-sweetened beverages	NA^e^	NA
**Foods served**		
Refined grains with trans fat	1 [Reference]	NA
Refined grains without trans fat	0.07 (0.02)	.003
Whole grains with trans fat	NA^e^	NA
Whole grains without trans fat	0.03 (0.06)	.60
Canned or frozen vegetables	0.08 (0.03)	.01
Fresh vegetables	0.002 (0.04)	.97
Canned, dried, or frozen fruits	0.15 (0.04)	.002
Fresh fruits	0.32 (0.05)	<.001
Protein foods	0.21 (0.04)	<.001

Abbreviation: NA, not applicable.

a Complete after-school snack includes all foods and beverages served during the after-school snack period.

b Coefficient estimates from regression predicting prices of 1,294 complete daily snacks served at 32 YMCA after-school sites during the academic year in 2006. All prices are inflation-adjusted to December 2009 prices. Model *R* ^2^ = 0.47.

c These values can be interpreted as the average difference in price in a complete snack depending on whether it contained each of these various beverages or foods, adjusting for the metropolitan area of each YMCA association, the total number of foods served, and each of the other variables in the table simultaneously. For example, snacks containing 100% juice were on average $0.21 more expensive than snacks containing water, controlling for other variables in the model.

d Calculated by regressing each characteristic with price as the dependent variable, controlling for other variables in the model.

e Not served on any days.

**Table 4:** Repeatedly Served After-School Snacks at 32 YMCA After-School Sites (n = 9,787 Snack-Days), 2006-2008^a^^,^ b

**Food and Beverage Snack Combinations^c^^,^ d**	No. of Days Served	Average Price per Snack, $	Average Calories per Snack, kcal	Average Dietary Fiber per Snack, g
Fresh fruit, 1% or 2% milk, water	398	0.62	186.7	3.1
Applesauce, 1% or 2% milk, water	111	0.44	204.6	1.2
Canned pineapple in light syrup, 1% milk, water	86	0.49	168.0	1.0
Carrots, hummus, water	28	0.56	71.0	3.2
Frozen cherries, cheese, water	28	0.72	207.0	1.2
Apple, cheese slices, water	23	0.59	199.6	2.9
Applesauce, string cheese, water	16	0.53	183.0	1.5
Carrots, tuna salad, Wheat Thins, water	15	0.51	265.0	3.7
Trail mix, 1% or 2% milk, water	14	0.56	272.0	2.0
Whole-wheat bread, green peppers, turkey slices, water	11	0.49	91.0	2.9
Baby carrots, string cheese, water	9	0.65	158.0	2.5
Apple slices, whole-wheat bagel, tuna salad, water	7	0.73	311.0	4.0
Apricots, whole-wheat cinnamon raisin bread, water	7	0.27	127.0	2.7
Broccoli, carrots, ranch dressing, water	7	0.73	287.0	3.0
Craisins, string cheese, Wheat Thins, water	7	0.63	370.0	3.7
Peaches canned in light syrup, 1% milk, water	7	0.52	170.0	1.6
Apples, string cheese, water	5	0.59	149.5	2.3
Apple slices, cheese, whole-wheat crackers, water	5	0.64	348.5	4.3
Applesauce, popcorn, 1% milk, water	5	0.51	263.0	2.4
Broccoli, celery, ranch dressing, 1% milk, water	5	0.67	268.0	1.9
Carrots, celery, ranch dressing, cheese slices, water	5	0.74	325.5	2.5
Whole-wheat crackers, vegetables, water	5	0.40	138.7	4.4

Abbreviations: ESHE, Environmental Standards for Healthy Eating; CACFP, Child and Adult Care Food Program.

a "Repeatedly served" defined as at least 5 times over 9 consecutive weeks.

b Foods that met ESHE and CACFP guidelines and were priced at or below the 2009-2010 CACFP reimbursement rate of $0.74/snack. Prices were inflation-adjusted to December 2009 prices.

c We included tap water with each snack for $0.03/serving.

d All milk is unflavored milk.
